# Hantavirus-Driven PD-L1/PD-L2 Upregulation: An Imperfect Viral Immune Evasion Mechanism

**DOI:** 10.3389/fimmu.2018.02560

**Published:** 2018-12-03

**Authors:** Martin J. Raftery, Mohammed O. Abdelaziz, Jörg Hofmann, Günther Schönrich

**Affiliations:** Berlin Institute of Health, Institute of Virology, Charité–Universitätsmedizin Berlin, Humboldt-Universität zu Berlin, Berlin, Germany

**Keywords:** bystander activation, hantaviruses, viral immune evasion, PD-L1, PD-L2, PD-1, CD86

## Abstract

Viruses often subvert antiviral immune responses by taking advantage of inhibitory immune signaling. We investigated if hantaviruses use this strategy. Hantaviruses cause viral hemorrhagic fever (VHF) which is associated with strong immune activation resulting in vigorous CD8+ T cell responses. Surprisingly, we observed that hantaviruses strongly upregulate PD-L1 and PD-L2, the ligands of checkpoint inhibitor programmed death-1 (PD-1). We detected high amounts of soluble PD-L1 (sPD-L1) and soluble PD-L2 (sPD-L2) in sera from hantavirus-infected patients. In addition, we observed hantavirus-induced PD-L1 upregulation in mice with a humanized immune system. The two major target cells of hantaviruses, endothelial cells and monocyte-derived dendritic cells, strongly increased PD-L1 and PD-L2 surface expression upon hantavirus infection *in vitro*. As an underlying mechanism, we found increased transcript levels whereas membrane trafficking of PD-L1 was not affected. Further analysis revealed that hantavirus-associated inflammatory signals and hantaviral nucleocapsid (N) protein enhance PD-L1 and PD-L2 expression. Cell numbers were strongly reduced when hantavirus-infected endothelial cells were mixed with T cells in the presence of an exogenous proliferation signal compared to uninfected cells. This is compatible with the concept that virus-induced PD-L1 and PD-L2 upregulation contributes to viral immune escape. Intriguingly, however, we observed hantavirus-induced CD8+ T cell bystander activation despite strongly upregulated PD-L1 and PD-L2. This result indicates that hantavirus-induced CD8+ T cell bystander activation bypasses checkpoint inhibition allowing an early antiviral immune response upon virus infection.

## Introduction

The immune response to infection is regulated not only by signaling through antigen receptors but also by co-receptors (1). The principal stimulatory co-receptor CD28 is constitutively expressed on T cells and interacts with CD80 and CD86 expressed on activated professional antigen-presenting cells (APCs) such as dendritic cells (DCs) (2). In contrast, programmed death-1 (PD-1), a member of the CD28 family, is a key negative regulator of immune responses (3). PD-1 is expressed on activated T cells whereas the known PD-1 ligands, PD-L1 and PD-L2, are detected on professional APCs similar to CD80 and CD86 (4). In addition, PD-L1 is expressed by non-hematopoietic cells such as endothelial cells (5–8). PD-L1 is further upregulated by proinflammatory cytokines that are released during virus infections such as type I and type II interferon (9). These pro-inflammatory cytokines also enhance PD-L2 expression, which is usually expressed at only low levels by a restricted number of cell types such as dendritic cells (DCs) (9).

Viruses have evolved mechanisms to exploit host inhibitory receptor signaling for subversion of host immune responses (10). Persisting viruses such as human immunodeficiency virus type 1 (HIV-1), hepatitis B virus (HBV) and hepatitis C virus (HCV) drive virus-specific CD8+ T cells into a dysfunctional or “exhausted” phenotype that is characterized by increased PD-1 expression (11, 12). In accordance, blockade of PD-1 or its ligands in chronic viral infection can enhance virus-specific CD8^+^ T-cell responses and reduce the viral load. The functional consequences of PD-L1 upregulation during acute viral infection are less clear (13). For example, CD8+ T cell responses are impaired and immunopathology is attenuated by the PD-1 pathway during acute virus infections of the lower respiratory tract (14). On the other hand, it has been reported that PD-L1 upregulation on DCs contribute to the antiviral defense during acute HSV-1 infection (15). Moreover, during acute Friend retrovirus infection CD8+ T cells expressing high levels of PD-1 were both cytotoxic and critical for virus control (16).

Viral hemorrhagic fever (VHF) is a term for a group of similar but distinct zoonotic human diseases that are caused by RNA viruses including hantaviruses. Humans are infected with hantaviruses after inhalation of aerosols that contain virions derived from the natural host reservoirs, mostly rodents (17). The hallmarks of VHF are increased vascular permeability and loss of platelets (18). Hantaviruses are known to replicate without causing obvious cytopathic effects. As with other VHFs dysregulated immune responses play a role in hantavirus-associated diseases (19, 20). Paradigmatic experiments with lymphocytic choriomeningitis virus (LCMV)-infected mice have shown that PD-L1 is critical for prevention of immunopathology and virus-induced dysfunction such as vascular leakage (21, 22). Thus, it is important to understand how hantavirus replication modulates PD-L1 and PD-L2. In this study, we investigated how hantavirus replication affects the key stimulatory and inhibitory checkpoints of immune responses and explored the functional consequences thereof.

## Materials and Methods

### Ethics Statement

The analyses of human sera were in accordance with the ethical standards of the institutional research committee and with the 1964 Helsinki declaration and its later amendments or comparable ethical standards. For this retrospective study, formal consent is not required. Buffy coat preparations were purchased from German Red Cross (Dresden). Blood samples were taken with the approval of the ethics committee of the Charité–Universitätsmedizin Berlin. Written informed consent was obtained from all donors.

### Cells

Vero E6 and RPE-1 cells were cultured in Dulbecco's MEM (Gibco) supplemented with 10% hiFCS (BioWhittaker), 2 mM L-glutamine, penicillin and streptomycin (PAA). HUVECs were generated and cultivated as described (23). Adherent cells were passaged by first washing with PBS (Biochrom), addition of trypsin until cells detached and finally addition of FCS-containing medium to stop trypsin. HEL cells, an erythroleukemia suspension cell line, were cultured in RPMI 1640 (Gibco) with 10% hiFCS, 2 mM L-glutamine, penicillin and streptomycin (PAA). Huh7.5 cells is a human hepatoma cell line, which expresses an endogenous RIG-I with a mutation (T55I) in the first caspase-recruiting domain. This mutated RIG-I acts as a dominant-negative inhibitor (24). Transduced Huh7.5 clones overexpressing constitutive active RIG-I have been generated previously and were cultured as described (25). Huh7.5 cells were cultured as previously described (26).

Density gradient centrifugation using Ficoll-Paque was used to isolate PBMCs from buffy coat units (DRK, Dresden). In short, blood diluted 1:1 with RPMI wash (RPMI 1640, 2% heat-inactivated FCS and 0.2 mM EDTA) was layered onto Ficoll (PAA) and centrifuged at 800 *g*, 30 min RT. PBMC were isolated from the interface, washed twice and CD14+ cells isolated using Blood CD14 isolation kit (Miltenyi Biotec). CD14+ monocytes were used to generate immature DCs by cultivation in RPMI1640 with 10% hiFCS (Hyclone), 2 mM L-glutamine, penicillin and streptomycin (PAA) and further supplemented with 500 IU/ml GM-CSF (ImmunoTools) and 200 IU/ml IL-4 (ImmunoTools). Medium and cytokines were changed every 2–3 days, cells were used for experiments at day 6.

### Cytokines And Pathogen-Associated Molecular Patterns (PAMPs)

IFN-α, IFN-β, and IFN-γ were provided by ImmunoTools. Further samples of IFN-β were supplied by R&D Systems. TLR3 agonist polyinosinic:polycytidylic acid [poly(I:C)] and polydeoxyadenylic:polydeoxythymidylic acid [poly(dA:dT)], which indirectly stimulates retinoic acid–inducible gene I (RIG-I), were obtained from InvivoGen. Poly(I:C) was used at 10 μg/ml and poly(dA:dT) at 1 μg/ml.

### Serum Samples And ELISAs

Samples from patients infected with Puumala virus (PUUV) or Dobrava-Belgrade virus (DOBV) were collected for diagnostic purposes and were anonymized and stored before being tested retrospectively. Routine diagnostic testing included qPCR of the L segment of hantavirus from RNA isolated from the sera, positivity indicating the presence of active viral infection and thus an acute infection. All serum samples were stored at −80°C before use. The histone/dsDNA complexes were determined using Cell Death Detection ELISA^PLUS^ (Roche) for quantification of neutrophil extracellular traps (NETs) in the serum as previously described (27). Human sPD-L1 and sPD-L2 levels were determined by using ELISA kits from R&D Systems, whereas the ELISA for measuring soluble CD86 (sCD86) was provided by PromoKine.

### Flow Cytometry of Surface Molecules

Cells were harvested and washed twice in ice-cold FACS washing solution. Cells were then resuspended in 50 μl FACS blocking solution, containing the primary antibody in appropriate dilution, and incubated for 1 h. Cells stained with directly-coupled antibodies were washed and analyzed. For uncoupled primary antibodies after incubation cells were again washed twice with FACS wash and secondary antibody, diluted in FACS block solution, was added. After 45 min the cells were washed with FACS wash solution and resuspended in FACS fixation solution. For quantifying fluorescence of labeled cells a FACSCalibur^®^ (BD Biosciences) was used. Results were evaluated with the flow cytometry analysis software programs CellQuestPro^®^ and FlowJo V10 (BD Biosciences).

### Transfection

Transfection was undertaken using plasmid pcDNA3.1 HTNV N or empty pcDNA3.1 as control (1 μg) using Lipofectamine 3000 transfection reagent according to the manufacturer's protocol, including Optimem medium (Thermo Fischer Scientfic).

### Antibodies And Staining Reagents

For flow cytometry and functional studies, respectively, the following antibodies and staining reagents were used: anti-CD40 (clone 5C3), anti-CD54 (clone HA58), anti-CD80 (L307.4), anti-CD83 (clone HB15e), anti-CD107a (H4A3), and anti-B7-H2 (clone 2D3) were supplied by BD PharMingen; anti-PD-1 (clone J116), anti-PD-L1 (clone MIH1), and anti-PD-L2 (clone MIH18), anti- B7-H3 (clone H74), anti-B7-H4 (clone MIH35) were purchased from eBioscience; anti-CD86 (clone IT2.2) was supplied by ImmunoTools; anti-DC-SIGN (Clone MR-1) was purchased from Acris; anti-MHC class I (clone w6/32) and II (clone L243) were produced in-house; HCMV pp65 495-503 loaded NTA HLA-A2 tetramer reagents were obtained from TCMetrix. Secondary antibodies coupled to fluorochromes were supplied by Dianova. Blocking monoclonal antibodies directed against human IL-15 (clone 34559) and anti-human IFNR chain 2 (clone MMHAR-2) were supplied by R&D Systems. Cells were incubated with blocking antibodies or isotype-matched control antibodies for 1 h before exposure to virus. Isotype-matched control antibodies were supplied by BD PharMingen. For immunohistochemistry human-specific FITC-coupled anti-MPO (clone 7.17; ImmunoTools) and polyclonal goat anti human PD-L1 (R&D Systems) were used with bovine anti goat Fab fragment Alexa 594-coupled (Dianova) as secondary antibody, all used at 1:300 dilution.

### PD-L1 Uptake Protocol

Cells were incubated with PE-coupled anti-PD-L1 antibody for 1 h at 4°C or 37°C for 4 h before being washed and analyzed by flow cytometry. Uptake was calculated by subtracting MFI at 37°C from MFI at 4°C. Uptake of HTNV infected cells was then compared to uninfected cells.

### T Cell Assay

CD4+ cells were isolated from PBMCs using CD4-coupled beads (Miltenyi) and frozen on liquid nitrogen until use. HUVECs infected with HTNV at a MOI of 1.5 were incubated in flat-bottom 96-well plates for 4 days before being were mixed with allogenic CD4+ cells at a ratio of 1:4 and treated with PHA at 5ug/ml for 2 days. Proliferation was measured by MTT dye test (EZ4U-test).

### Viruses

Virus stocks of Hantaan virus (HTNV, strain 76-118) and Tula virus (TULV, strain Moravia) were propagated on VeroE6 cells in a biosafety level 3 (BSL3) laboratory as previously described (28). For virus titration, supernatants from hantavirus-infected cells were incubated with Vero E6 cells and subsequently focus-forming units (FFU) were counted in a chemiluminescence detection assay (29). Virus stocks were regularly tested for mycoplasma by PCR and stored at −80°C before use. In order to infect cells virions were allowed to adsorb to cells for 1 h. After infection cells were washed three times with medium before incubation in a humidified incubator at 37°C. Uninfected cells treated with medium instead of virions were used as mock control. Herpes simplex virus type 1 (HSV-1) strain KOS and Vesicular stomatitis virus (VSV, strain Indiana) was propagated and titrated as previously described. Titres were determined by plaque assay on Vero E6 cells and expressed as PFU per milliliter (30). UV inactivation was performed for 5 min and the remaining titer was tested and found to be less than 1 FFU/ml.

### qPCR

RNA was isolated from cells using RNeasy Plus mini kit (Quiagen) and reverse transcribed using SuperScript III (Thermo Fisher Scientific). qPCR was performed on a qTOWER^3^ (Analytik Jena) using PrimeTime gene expression master mix and PrimeTime primers (IDT). The input RNA was normalized using average expression of β-actin and cyclophilin B housekeeping genes.

### Humanized Mouse Model

The generation of mice with a humanized immune system has been described elsewhere (31). Briefly, NSG mice expressing HLA-A2, a human MHC class I molecule, were humanized by reconstitution with HLA-A2+ human CD34+ hematopoietic stem cells isolated from umbilical cord blood. Engraftment was evaluated at 11 weeks post inoculation by cytofluorimetric analysis of PBMCs. Successfully engrafted mice were infected i.p. with 10^5^ focus-forming units (FFU) of HTNV (strain 76-118). Infection was successful as determined by qPCR from sera. Twenty-Two days post infection mice were sacrificed and liver, kidney, lungs and spleen fixed and mounted in paraffin blocks. The infection experiments were approved by the governmental animal-welfare committee of the state Berlin, Germany (G 0013/12).

### ImageJ Analysis

Six cell-rich areas of five to twelve cells each were analyzed on each slide. Cell density was determined blind using DAPI staining and subsequently the mean intensity of staining of human PD-L1 (Texas Red) was determined.

### Statistical Analysis

Student's *t*-test and 1 way ANOVA test with Bonferroni correction were used to determine statistical significance. *P-*values below 0.05 (95% confidence) were considered to be significant. Prism 6 software (GraphPad) was used for statistical analysis.

## Results

### Strong Upregulation of PD-1 Ligands in Hantavirus-Infected Patients and in an Animal Model of Hantavirus Infection

**Figure 1:**
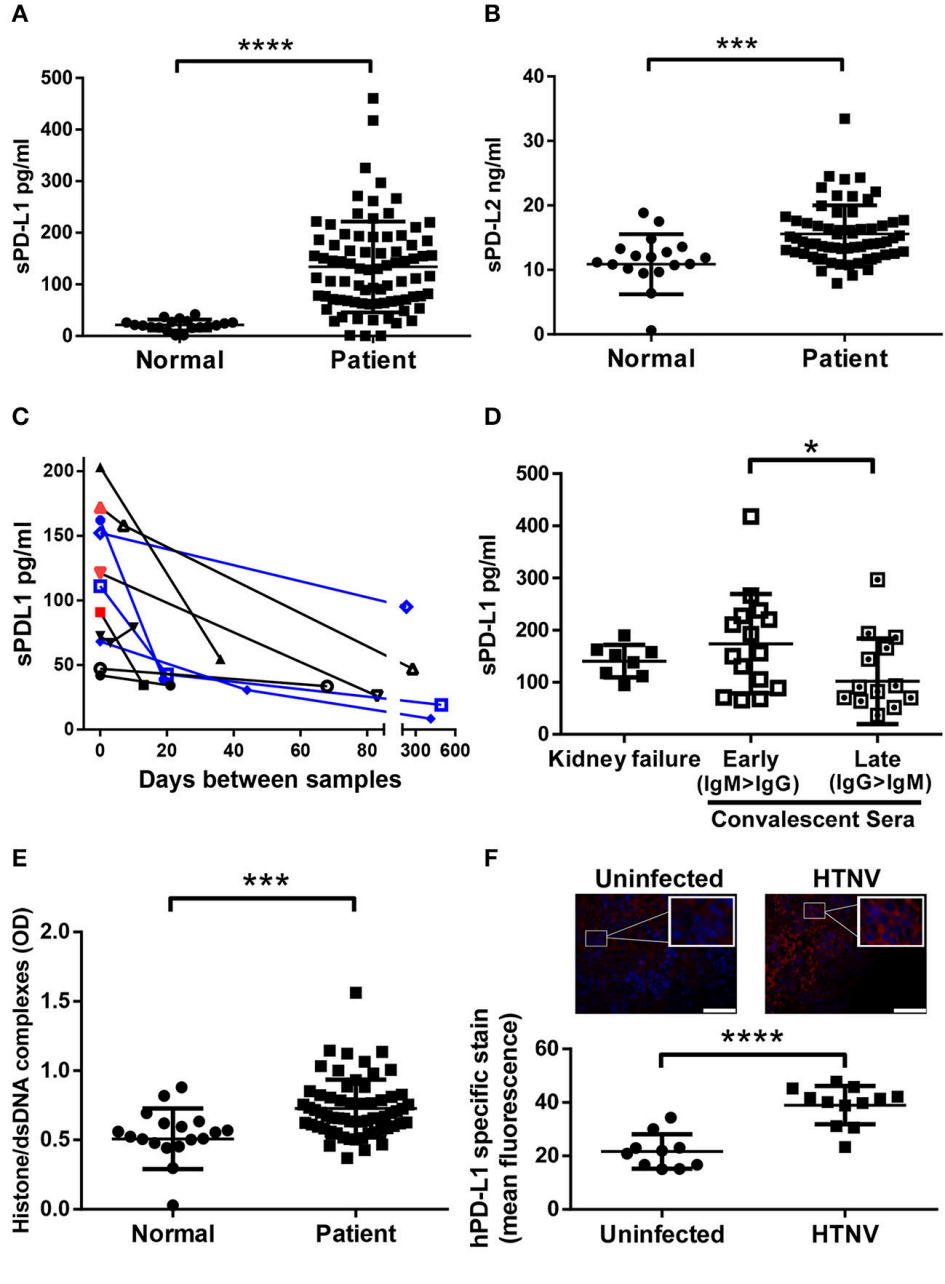
Levels of sPD-L1, sPD-L2, hantavirus-specific IgG and NETs in sera from hantavirus-infected patients. Sera from normal healthy individuals or convalescent hantavirus-infected patients (after the viremic phase) was tested by ELISA for levels of **(A)** sPD-L1 and **(B)** sPD-L2. Error bars represent the mean ± SD (^****^p < 0.0001, ^***^p < 0.001, paired Student's t-test). **(C)** Sequential sera samples from patients with PUUV (black) or DOBV (blue) were tested for sPD-L1. Red samples also tested additionally positive for hantavirus RNA and are therefore acute infections. **(D)** Levels of sPD-L1 in patients with kidney failure or in convalescence were further analyzed. Convalescent sera were separated into early convalescent (IgM dominant) or late convalescent (IgG dominant). Error bars represent the mean ± SD (^*^p < 0.05, paired Student's t-test). **(E)** The level of NETs in sera from normal healthy individuals or convalescent hantavirus-infected patients was determined as previously described (27). Error bars represent the mean ± SD (^***^p < 0.001, paired Student's t-test). **(F)** Spleen sections from uninfected or HTNV-infected humanized mice were stained for human PD-L1 (red) and nuclei (blue). HTNV-infected spleen sections show large areas of human cells with enhanced PD-L1 expression in comparison to uninfected spleen sections (upper left and right panel; inserts show higher magnification of cells; bars represent 100 μm). Slides from uninfected and HTNV-infected humanized and unreconstituted mice animals (N = 3 each group; 12 total) were analyzed using ImageJ to determine the intensity of human PD-L1 staining (Lower panel). Error bars represent the mean ± SEM (^****^p < 0.0001, paired Student's t-test). The samples from unreconstituted mice were used to determine the background staining. No significant difference was found in background staining in HTNV-infected or uninfected unreconstituted mice.

Initially we tested if hantaviruses modulate the expression of the ligands of checkpoint inhibitor PD-1 during clinical infection of humans. For this purpose, we measured the amount of soluble PD-L1 (sPD-L1) and soluble PD-L2 (sPD-L2) in sera from hantavirus-infected patients. The level of both sPD-L1 (Figure 1A) and sPD-L2 (Figure 1B) were strongly upregulated in sera from hantavirus-infected patients as compared to normal healthy individuals. Sequential samples from the same patients indicate that for both PUUV and DOBV sPD-L1 levels decrease with time indicating active regulation and that acute samples still with active virus replication (hantavirus RNA positive) have high sPD-L1 levels (Figure 1C). Similarly, PUUV samples early in convalescence (IgM > IgG) had significantly raised sPD-L1 compared to samples taken later (IgG > IgM) (Figure 1D). We also detected elevated levels of neutrophil extracellular traps (NETs), a marker for recent hantavirus infection, in these sera (Figure 1E) (27, 32). The level of sPD-L1 detected in culture supernatants and plasma of patients is known to correlate with the level of membrane-bound PD-L1 (33, 34). Taken together, PD-L1 and PD-L2 are strongly upregulated in hantavirus infected patients. Using a previously established animal model of hantavirus-induced immunopathology we analyzed the spleen of hantavirus-infected mice with a humanized immune system as previously published (31). We observed enhanced expression of human PD-L1 in the spleen (Figure 1F) in addition to high levels of human myeloperoxidase (MPO)-expressing cells, presumably neutrophils (data not shown). Taken together this data shows that PD-L1 and PD-L2 are strongly upregulated during hantavirus infection *in vivo*.

### Hantavirus-Infected Human Dendritic Cells Upregulate Both Costimulatory Molecules as Well as PD-L1/PD-L2

**Figure 2:**
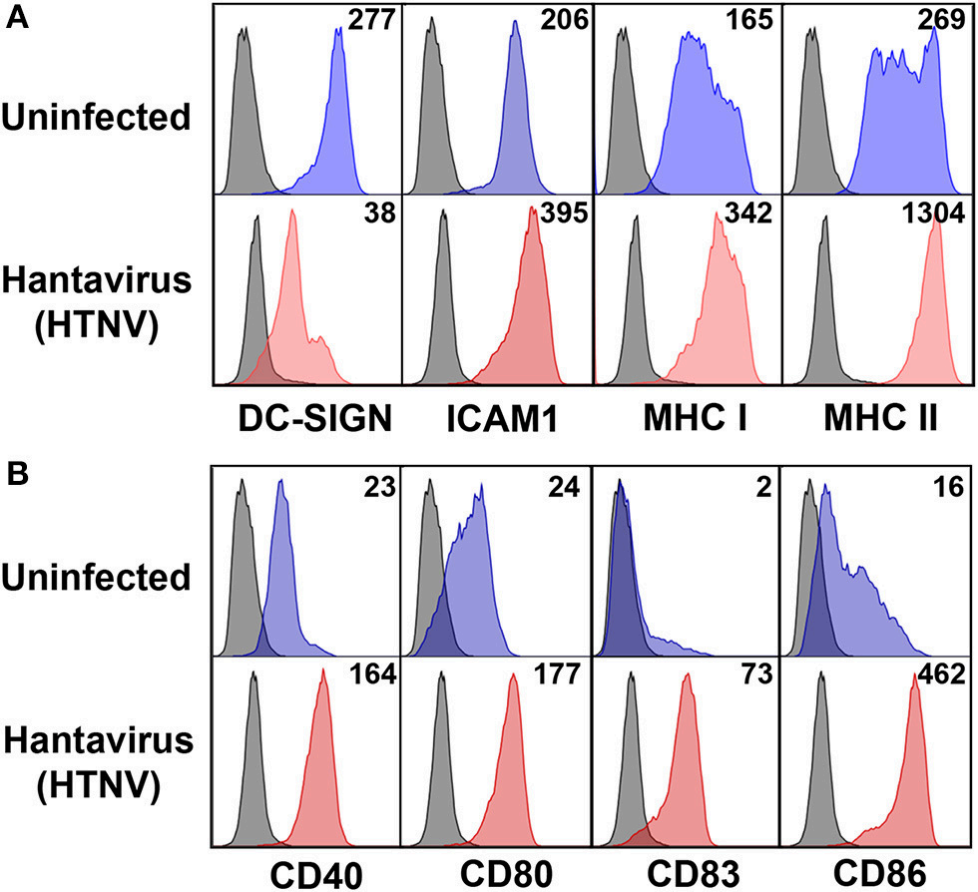
Mature DC phenotype after hantavirus infection. Immature DCs were infected with HTNV at MOI of 1.5 and incubated for 4 days before staining for **(A)** maturation markers and **(B)** costimulatory markers. The results shown are representative of three independent experiments using three different donors.

**Figure 3:**
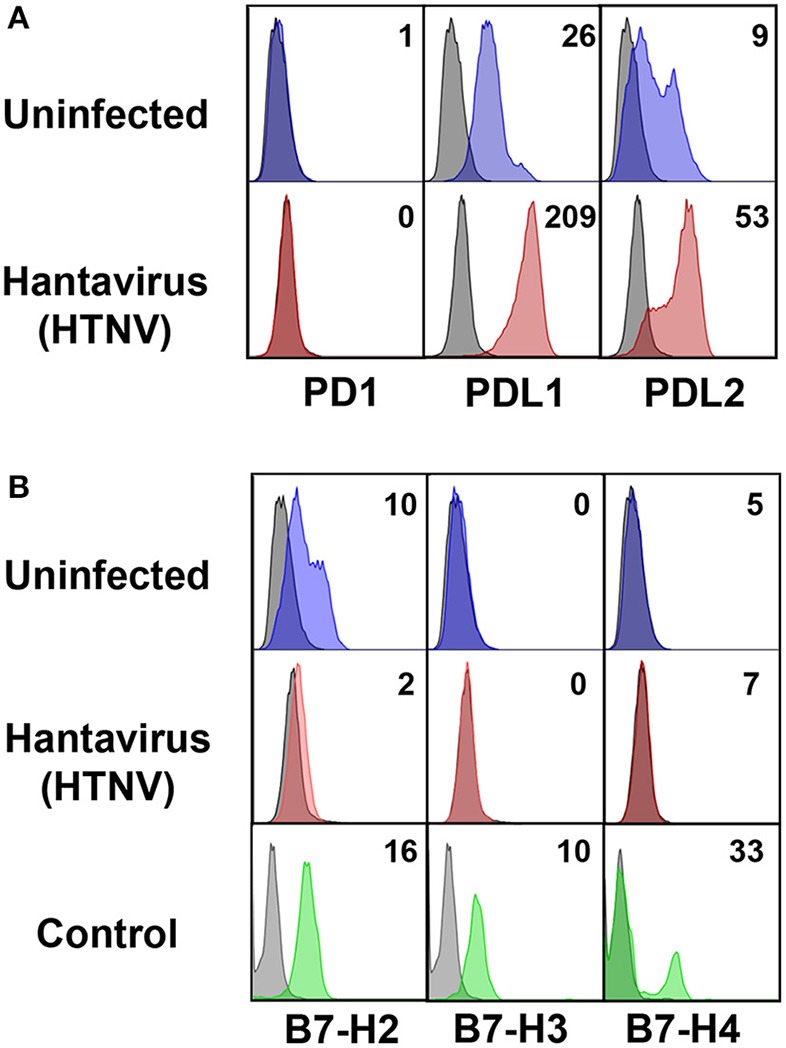
Hantavirus-induced upregulation of PD-L1 and PD-L2 on immature DCs. **(A)** Immature DCs were infected with HTNV at a MOI of 1.5 and incubated for 4 days before staining for PD-1, PD-L1 or PD-L2. **(B)** Immature DCs infected as for **(A)** were stained for members of the B7 family other than PD-L1/PD-L2. The results shown are representative of three independent experiments using three different donors. Positive controls are given in the lower panel (B7-H2 and B7-H3 from HUVEC, B7-H4 from HEK293 cells transfected with a B7-H4 plasmid).

Next we investigated the possible source of sPD-L1 and sPD-L2 seen in sera from hantavirus-infected patients. The production of sPD-L1 by proteolytic cleavage of membrane-bound PD-L1 is a feature of activated monocyte-derived DCs (35). This important immunoregulatory cell type is susceptible to hantavirus infection (36–39). As previously reported, immature DCs infected with Hantaan virus (HTNV), the most common cause of human hantavirus infections, upregulated adhesion molecules and MHC molecules (Figure 2A). In addition, HTNV increased expression of costimulatory molecules on the surface of immature DCs (Figure 2B). Intriguingly, HTNV infection resulted in enhanced expression of both PD-L1 and PD-L2 whereas PD-1 was barely detectable on the surface of uninfected and HTNV-infected immature DCs (Figure 3A). In contrast, HTNV-infected DCs did not upregulate other members of the B7 family such as B7-H2, B7-H3, and B7-H4. (Figure 3B) (40). In summary, hantavirus replication in DCs drives surface expression of both T cell costimulatory molecules such as CD86 as well as the T cell inhibitory molecules PD-L1/PD-L2.

### Hantavirus Regulates PDL1/PDL2 Expression on the Transcription Level

**Figure 4:**
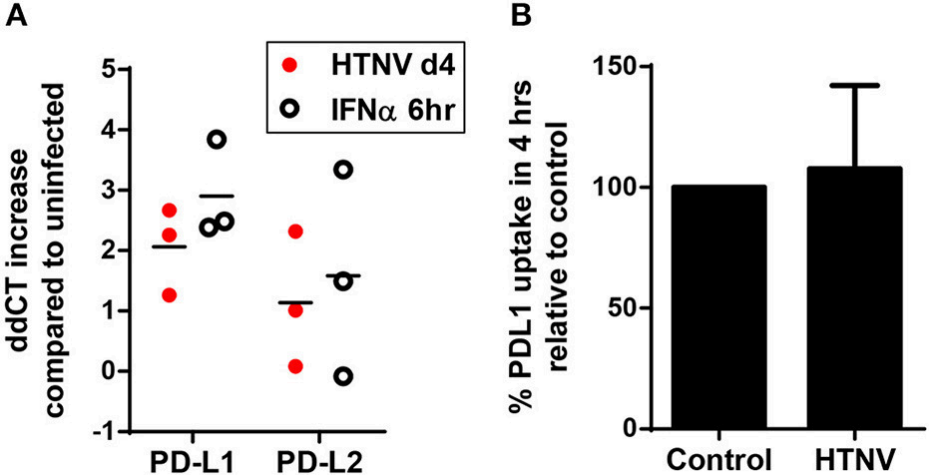
Increase in PD-L1 and PD-L2 transcripts but not cellular uptake in hantavirus-infected immature DCs. **(A)** Immature DCs were infected with HTNV at MOI of 1.5 and incubated for 4 days or exposed to IFN-α for 6 h at 2,000 U/ml before being harvested. Subsequently, RNA was isolated and the number of indicated transcripts quantified by qPCR according to the delta-delta-Ct (ddCt) method. **(B)** Immature DCs infected as for **(A)** were incubated with PE-coupled anti-PD-L1 antibody at 4°C 1 h or at 37°C for 4 h before being washed and analyzed by flow cytometry. Uptake was calculated by subtracting MFI at 37°C from MFI at 4°C. Uptake of HTNV infected cells was then compared to uninfected cells. Results are derived from three independent experiments, error bars represent the mean ± SD.

In further experiments we analyzed the mechanism upregulating PD-L1 and PD-L2 during hantavirus infection of DCs. PD-L1 expression can be regulated on the genetic, transcriptional, post-transcriptional and post-translational level (41). We first determined the number of PD-L1 and PD-L2 transcripts in HTNV-infected DCs and DCs exposed to IFN-α by qPCR. HTNV increased the number of transcripts encoding PD-L1 and PD-L2 (Figure 4A). IFN-α also upregulated PD-L1 and PD-L2 transcripts. We also tested whether HTNV modulates DCs trafficking of PD-L1. As shown in Figure 4B HTNV-infected DCs endocytosed PD-L1 as efficiently as uninfected control cells excluding altered endocytosis kinetics as a mechanism of PD-L1 upregulation. In conclusion, hantaviruses increase the number of PD-L1/PD-L2 transcripts but do not modulate endocytosis of the corresponding proteins.

### Hantavirus-Associated Inflammatory Signals Including Hantaviral N Protein Drive PD-L1 Expression

**Figure 5:**
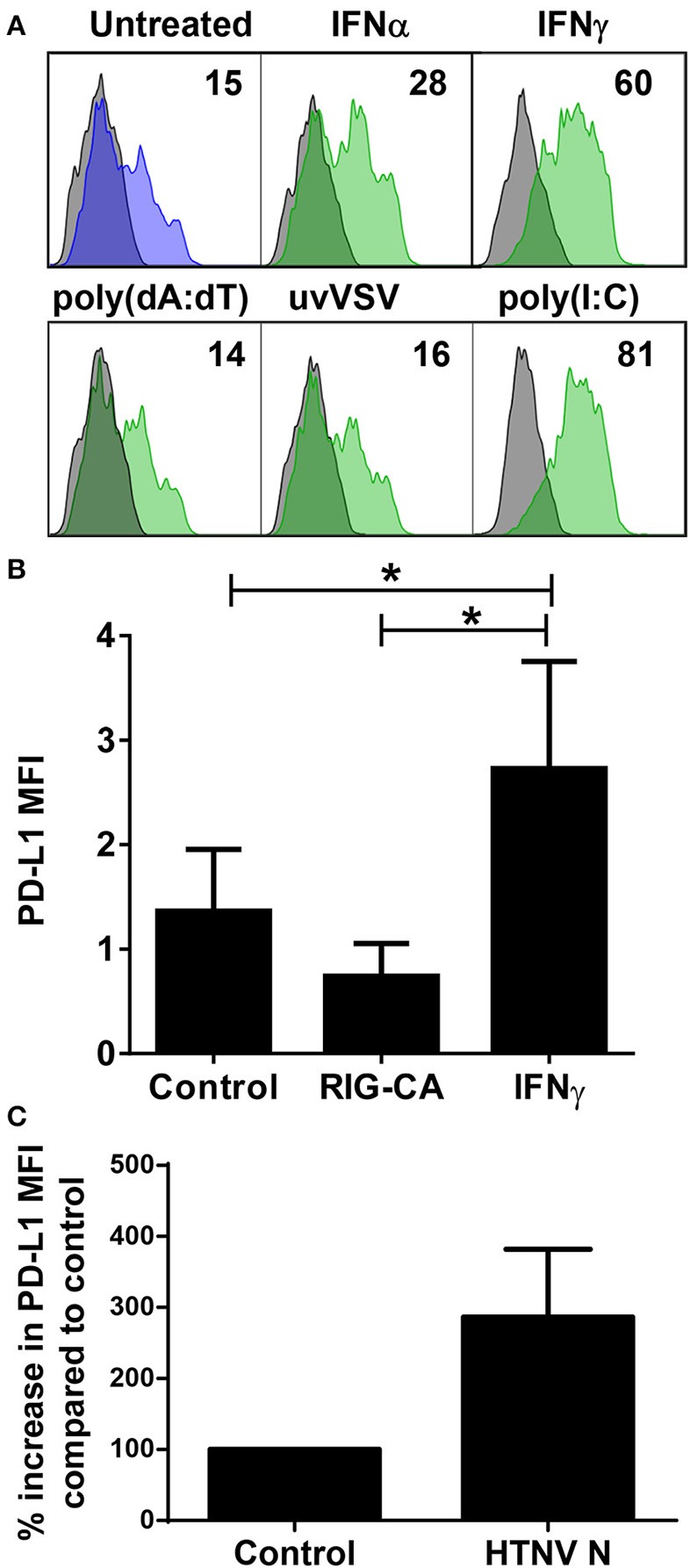
Control of PD-L1 expression by inflammatory stimuli. **(A)** Immature DCs were exposed to the following inflammatory stimuli before staining for PD-L1: type I IFN (IFN-α at 1,000 U/ml), type II IFN (IFN-γ at 1,000 U/ml), poly(dA:dT), UV-inactivated VSV or poly(I:C) for 24 h. The results shown are representative of three independent experiments using three different donors. **(B)** Huh7.5 cells (control), Huh7.5 cells permanently expressing a constitutively active form of RIG-I (RIG-CA) or Huh7.5 cells stimulated with IFN-γ at 1,000 U/ml for 24 h were stained for PD-L1 and analyzed by flow cytometry. Results are derived from three independent experiments, error bars represent the mean ± SEM (^*^p < 0.05, paired Student's t-test). **(C)** HEL cells were transfected with HTNV N-expressing plasmids or empty plasmids (Control). After 2 days cells were stained for PD-L1. Results are given as a percentage of control and are derived from three independent experiments, error bars represent the mean ± SD.

Next we examined which hantavirus-associated inflammatory stimuli modulate PD-L1 expression on immature DCs. IFN-γ and to a lesser extent IFN-α upregulated cell-surface PD-L1 (Figure 5A). Hantavirus replication triggers pattern recognition receptors (PRRs) such as toll-like receptor 3 (TLR3) and retinoic acid–inducible gene I (RIG-I) (30, 42, 43). Strikingly, TLR3 agonist poly(I:C) strongly increased PD-L1 expression on immature DCs (Figure 5A). Poly(I:C) similarly induced PD-L2 (data not shown). In contrast, immature DCs treated with RIG-I activating signals such as UV-inactivated VSV or poly(dA:dT) did not show increased PD-L1 expression (Figure 5A). The absence of PD-L1 upregulation after stimulation of the RIG-I pathway was confirmed by using Huh7.5 cells expressing a constitutive active RIG-I molecule (RIG-CA) (25). These cells did not express elevated PD-L1 levels compared to the untreated cells whereas Huh7.5 cells treated with IFN-γ upregulated PD-L1 compared to untreated Huh7.5 cells (Figure 5B). We also tested the effect of hantaviral nucleocapsid (N) protein, which has many diverse functional activities during the viral life cycle (44). As shown in Figure 5C expression of N protein in HEL cells, a human erythroleukemia cell line, resulted in PD-L1 upregulation. In summary, type I IFN, hantaviral N protein, and TLR3 signaling induced PD-L1 expression whereas RIG-I signaling had no effect.

### Subversion of T Cell Responses by Hantavirus-Induced Checkpoint Inhibitors

**Figure 6:**
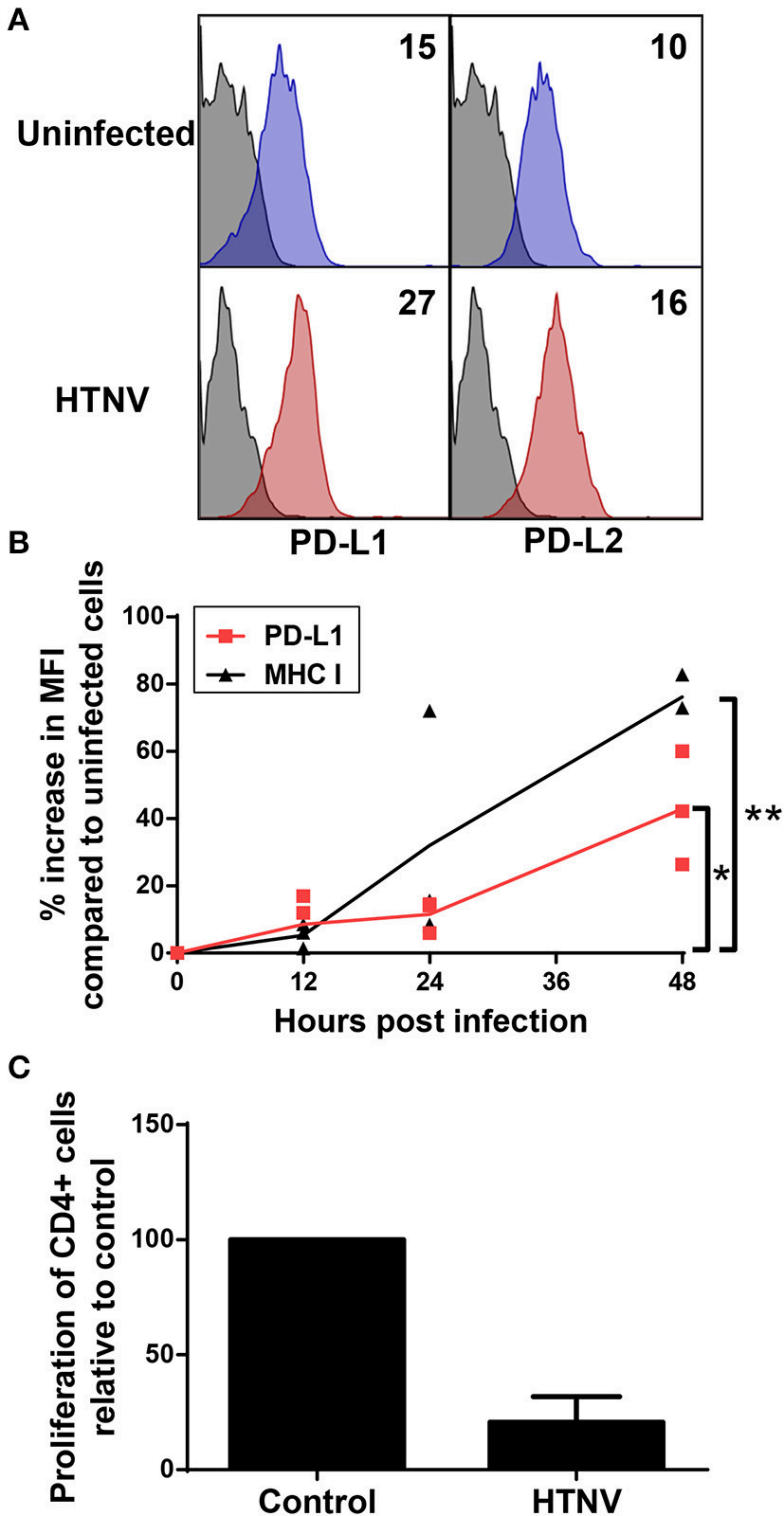
Upregulation of functional PD-L1 and PD-L2 on HTNV-infected endothelial cell lines. **(A)** HUVECs were infected with HTNV at a MOI of 1.5 and incubated for 4 days before staining for PD-L1 or PD-L2. The results shown are representative of 4 independent experiments using 4 different donors. **(B)** Human primary fibroblasts (Fi301) cells were infected at a MOI of 1.5 and incubated for 12, 24 or 48 h before staining for PD-L1 or MHC class I molecules. Results are derived from three independent experiments (^*^p < 0.05, ^**^p < 0.01, paired Student's t-test). **(C)** HUVECs infected as for **(A)** were mixed with allogeneic CD4+ cells at a ratio of 1:4 and treated with PHA at 5 μg/ml. After 2 days the number cells was measured by MTT dye test (EZ4U-test). Results are derived from three independent experiments using three different donors, error bars represent the mean ± SD.

We next analyzed whether PD-L1 and PD-L2 is upregulated on hantavirus-infected endothelial cells, which play a pivotal role in hantavirus pathogenesis (45, 46). Upon hantavirus infection human umbilical vein endothelial cells (HUVECs) upregulated both PD-L1 and PD-L2 (Figure 6A). PD-L1 expression started to increase on HTNV-infected cells at 12 h post infection similar to MHC class I expression (Figure 6B). PD-L1 expression further increased at later time points post infection (Figure 6B). We also tested whether hantavirus-induced PD-L1 and PD-L2 modulate T cell responses. For this purpose, HTNV-infected HUVECs were mixed with allogeneic CD4+ cells and stimulated with PHA. T cells strongly upregulate PD-1 upon stimulation with PHA (47). As shown in Figure 6C the numbers of surviving T cells and endothelial cells was strongly reduced in comparison to control T cells exposed to uninfected HUVECs, suggesting that T cell proliferation may be reduced. These results indicate that hantaviruses upregulate both PD-L1 and PD-L2 on endothelial cells which has a functional effect on T cells.

### Hantavirus-Induced Bystander Activation Despite Upregulation of Checkpoint Inhibitors

**Figure 7:**
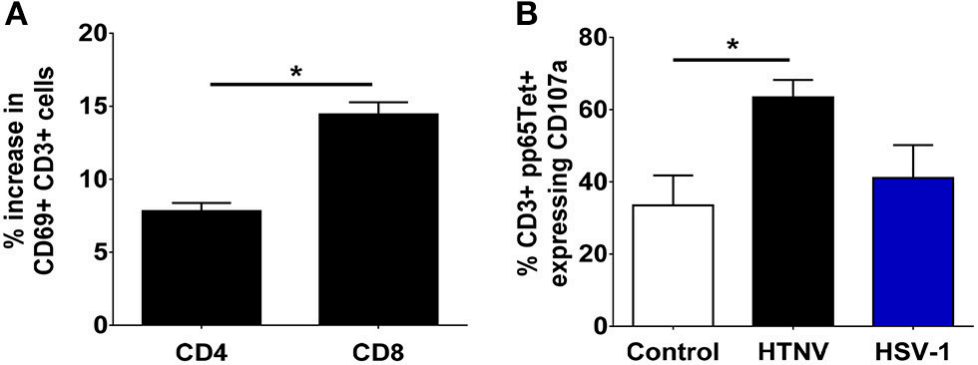
Monocyte-dependent bystander activation of CD8+ T lymphocytes by hantavirus. PBMCs isolated from blood of healthy human donors were mock-infected or infected with HTNV. After 3–4 days cells were analyzed by flow cytometry. **(A)** HTNV-specific increase in CD69+ cells in the CD4+ and CD8+ subset of CD3+ cells after 4 days of incubation. Results are from three independent experiments. Error bars represent the mean ± SEM (^*^p < 0.05, paired Student's *t-*test). **(B)** PBMCs from HLA-A2+ HCMV-seropositive healthy human donors were exposed to HTNV or HSV-1 for 4 days before being stained for HCMV-specific CD3+ cells using a pp65 loaded tetramer reagent (CMVpp65TET). Degranulation was determined by CD107a staining. Results are derived from three independent experiments. Error bars represent the mean ± SEM (^*^p < 0.05, paired Student's *t-*test).

To test the functional consequences of PD-1 ligand upregulation we investigated the behavior of T cells when exposed to infected autologous myeloid cells. We infected PBMCs from healthy human donors with HTNV and subsequently stained T cells for expression of the C-type lectin CD69 as an early marker of T-cell activation (48). Recently, it has been shown that CD69 regulates the metabolism and migration-retention ratio of T cells as well as the acquisition of T cell effector or regulatory phenotypes (49). Surprisingly, we observed increased percentages of activated cells especially in the CD8+ T cell population early after infection of PBMCs with HTNV (Figure 7A). Bystander activation of T cells during viral infections is common and is initiated by stimulated professional APCs such as DCs (50). In order to identify the responding cells, we tested whether heterologous memory CD8+ T cells are activated in this experimental setting. For this purpose we infected PBMCs derived from HLA-A2+ human healthy donors that were seropositive for human cytomegalovirus (HCMV), a member of the human herpesvirus family. A HLA-A2 tetramer loaded with a immunodominant peptide derived from pp65 (CMVpp65TET) was used to detect HCMV-specific CD8+ memory T cells. After HTNV infection of PBMCs the percentage of CMVpp65TET+ CD8+ T cells that expressed CD107a (LAMP-1), a marker for degranulation of activated CD8+ T cells (51), significantly increased in PBMCs as compared to uninfected PBMCs (Figure 7B). In contrast, in PBMCs infected with herpes simplex virus type 1 (HSV-1), another member of the human herpesvirus family, no significant increase in activated CMVpp65TET+ CD8+ T cells was observed (Figure 7B). In conclusion, heterologous T cells are activated at an early time point after hantavirus infection despite increased expression of PD-L1 on antigen-presenting cells.

### CD86-Dependency of Hantavirus-Induced Bystander Activation

**Figure 8:**
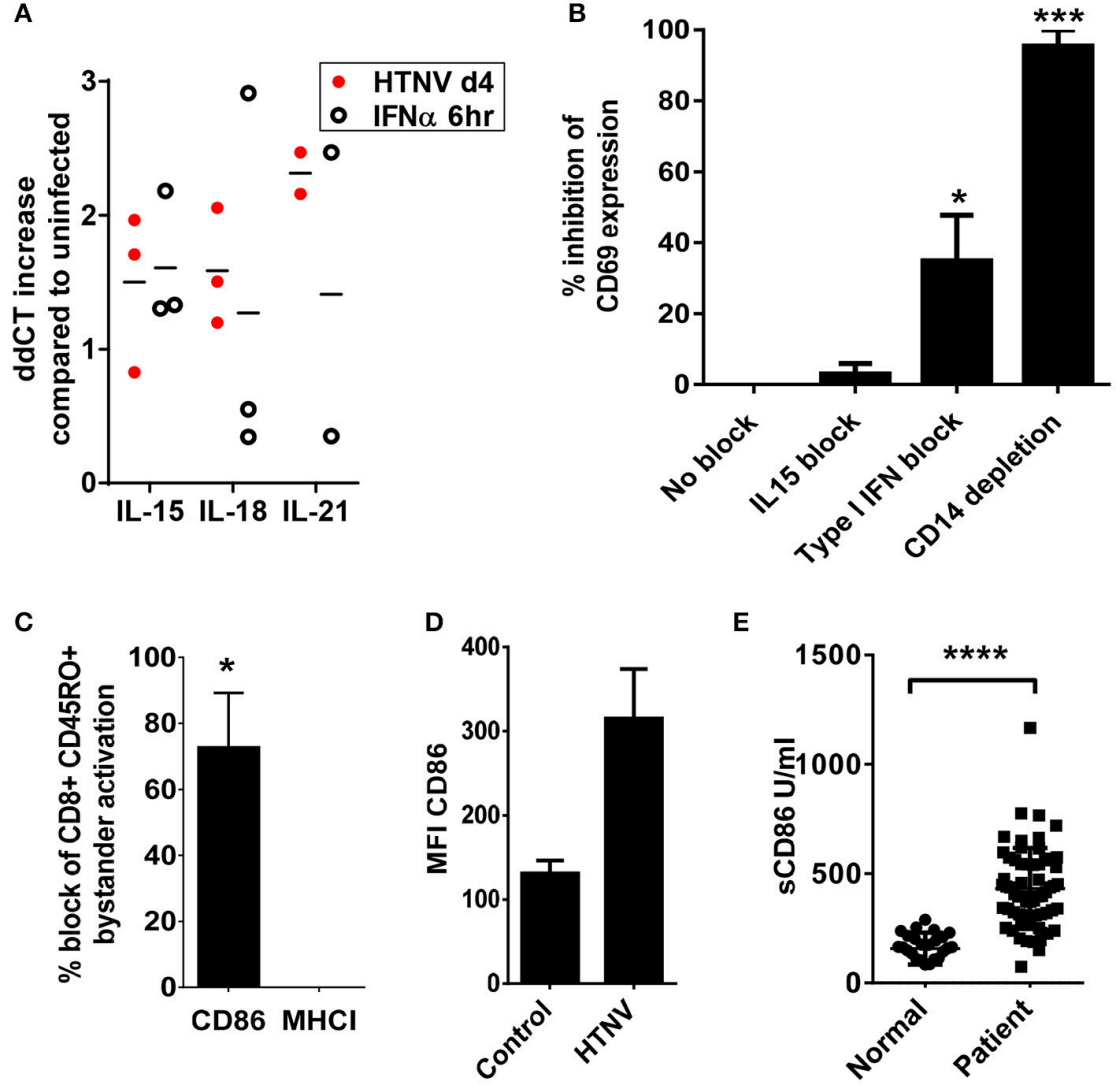
Dependency of hantavirus-induced bystander activation on costimulatory CD86 molecules. **(A)** Immature DCs were infected with HTNV at a MOI of 1.5 and incubated for 4 days or exposed to IFN-α for 6 h at 2,000 U/ml before being harvested. Subsequently, cellular RNA was isolated and the number of indicated cytokine-encoding transcripts quantified by qPCR according to the delta-delta-Ct (ddCt) method. **(B)** PBMCs treated with anti-IL15 (20 μg/ml) or anti-IFN-α (20 μg/ml) and PBMCs depleted of CD14+ cells were exposed to HTNV at a MOI of 1.5 for 4 days before CD69 expression on CD8+ cells was measured by cytofluorimetric analysis. Results are derived from three independent experiments, error bars represent the mean ± SEM (^*^p < 0.05, ^***^p < 0.001, 1 way ANOVA test with Bonferroni correction). **(C)** PBMCs treated with anti-CD86 or anti-MHC (both 10 μg/ml) were exposed to HTNV at a MOI of 1.5 for 4 days before CD69 expression on CD8+ CD45RO+ cells was determined by cytofluorimetric analysis. Error bars represent the mean ± SEM (^*^P < 0.05, Student's t-test). **(D)** PBMC infected with MOI 1.5 of TULV or HTNV were analyzed 3 days post infection for the expression of CD86 on the surface of CD14+ cells. **(E)** Sera from normal healthy individuals or convalescent hantavirus-infected patients were tested by ELISA for levels of sCD86. Error bars represent the mean ± SD (^****^p < 0.0001, paired Student's t-test).

We next examined the mechanisms by which hantavirus-infected DCs cause bystander activation despite checkpoint inhibitors. First, we tested whether hantavirus-infected DCs express inflammatory cytokines that can cause bystander activation of memory CD8+ T cells in the absence of cognate antigen such as IL-15, IL-18, and IL-21 (52). For this purpose RNA from immature DCs infected with HTNV or exposed to IFN-α was isolated and subjected to qPCR. As shown in Figure 8A HTNV upregulated production of mRNA encoding IL-15, IL-18, and IL-21 in immature DCs. This finding is in line with cytokine-drive bystander activation of T cells during hantavirus infection of PBMCs. In order to further dissect the mechanism we used antibodies to block IL-15 as this cytokine has been implicated in hantavirus-induced natural killer (NK) cell activation (53). We also blocked type I IFN, which also can contribute to bystander activation of T cells (52). The IL-15 block had no significant effect whereas the type I IFN block significantly reduced T cell bystander activation (Figure 8B). In comparison depletion of CD14+ cells completely abrogated hantavirus-induced bystander activation (Figure 8B). CD14 serves as marker for monocytes which are detected in PBMCs at frequencies of 10–20% (54) and represent the major hantavirus-permissive cell type in PBMCs. In addition, by blocking the T cell costimulatory molecule B7-2 (CD86) during HTNV infection of PBMCs we could also prevent bystander activation of CD8+ T cells (Figure 8C). In contrast, blocking of MHC class I molecules had no effect (Figure 8C). These result suggested that CD86 expressed by CD14+ cells plays a major role in hantavirus-induced bystander activation whereas interaction of T cell receptors (TCRs) with MHC-bound peptides interactions is not required (Figure 8C). In accordance, CD14+ cells strongly upregulated CD86 during infection with HTNV (Figure 8D) and high levels of soluble CD86 were detected in hantavirus-infected patients (Figure 8E). Taken together, these results demonstrate that CD14+ monocytes are inducing hantavirus-driven bystander T cell activation in a CD86-dependent manner.

## Discussion

In this study, we detected high amounts of sPD-L1 and sPD-L2 in sera of hantavirus-infected patients. Hantaviruses strongly upregulated PD-L1 and PD-L2 on endothelial cells, which play a pivotal role in hantavirus-induced pathogenesis. In line with an inhibitory role of PD-L1/PD-L2 hantavirus-infected endothelial cells did not induce T cell proliferation. Hantaviruses also strongly increased expression of PD-L1 and PD-L2 on monocyte-derived DCs. However, monocyte-derived inflammatory cells could still activate heterologous CD8+ T cells in a CD86-dependent fashion. This indicates that hantavirus-induced CD8+ T cell bystander activation bypasses inhibitory checkpoints.

Gene expression of PD-L1 and PD-L2 is controlled by inflammatory signals (9). Hantavirus-induced upregulation of PD-L1 and PD-L2 could be indirect due to release of IFNs. In line with this view, endothelial cells and DCs predominantly produce IFN-β upon infection with pathogenic hantaviruses (37, 38, 55, 56). PD-L2 is upregulated equally well by IFN-β and IFN-γ whereas PD-L1 is especially sensitive to IFN-γ (57). In hantavirus-infected patients vigorous responses of NK cells and CD8+ T cells resulting in increased levels of IFN-γ are observed (19, 58–61). In addition to this, we show that hantaviral N protein in HEL cells resulted in PD-L1 upregulation although the underlying mechanism is unclear. Thus, IFN-independent mechanisms may contribute to hantavirus-induced PD-L1/PD-L2 expression as recently shown for MHC class I molecules (62). In conclusion, PD-L1/PD-L2 upregulation in hantavirus-infected patients is due to both IFNs and additional IFN-independent mechanisms.

Hantavirus infection is detected by pattern recognition receptors, primarily TLR-3 (42) and RIG-I. (30, 43). We found that the TLR3 ligand poly(I:C) strongly increased PD-L1 levels on immature DCs. In accordance, poly(I:C) has been reported to upregulate PD-L1 on DCs (63, 64) as well as endothelial cells (65) and airway epithelial cells (66). In contrast, PD-L1 was not upregulated upon stimulating RIG-I. Taken together, our *in vitro* observations would fit with hantavirus infection strongly inducing PD-L1 and PD-L2 by triggering TLR-3, which transmits downstream signals through the TIR-domain-containing adapter-inducing IFN-β (TRIF) pathway. Production of IFN-β by both TLR3 and RIG-I induced signaling would be expected to further increase expression of PD-1 ligands later in infection.

Other viruses have also been reported to modulate checkpoint inhibitors. Similar to hantaviruses the Japanese encephalitis virus nonlytically infects monocyte-derived DCs thereby inducing phenotypic maturation and a significant increase in PD-L1 expression (67). Replication competent but not inactivated KSHV induces PD-L1 expression in human monocytes in a dose-dependent manner although the precise mechanism has not been defined (68). Akhmetzyanova et al. observed a type I IFN-dependent increase in PD-L1 expression after infection of spleen cells with the murine Friend retrovirus (FV) (69). PD-1 and PD-L1 are also up-regulated in monocytic cells upon HIV-1 infection (70, 71). In accordance, the HIV-1 Tat protein has been observed to increase PD-L1 expression on DCs through TNF-α and TLR4 (72). The HCV core protein up-regulates PD-L1 expression on Kupffer cells, which binds PD-1 to promote T cell dysfunction and development of viral persistence (73). A subset of macrophages upregulated PD-L1 expression via type I IFN during infection with LCMV (74). In addition, influenza virus enhances PD-L1 expression of lung macrophages through type I IFN signaling (75). Taken together, it appears that PD-L1 upregulation is a relatively common consequence of viral infection which is driven by type I IFN and viral PRR triggering.

PD-L1 expression on professional APCs facilitates the induction of regulatory T cells (Tregs) and enhances expression of the key transcription factor forkhead box p3 (Foxp3) (76, 77). Tregs not only regulate effector T cell function but also humoral immunity (78). A recent report has shown that the severity of hantavirus-associated disease correlates with expression of Foxp3 (79). This strongly suggests that hantavirus-induced upregulation of PD-L1 on DCs induces Tregs. In accordance, other investigators have shown that virus-induced PD-L1 upregulation on monocyte-derived DCs leads to expansion of Tregs (67).

We observed that hantavirus-infected human endothelial cells upregulate surface expression of PD-L1 and PD-L2 and inhibit proliferation of PHA-stimulated T cells. Other investigators detected increased amounts of PD-L1 in hantavirus-infected cultures of rat endothelial cells (80). In HFRS patients, hantavirus-induced PD-L1 may be responsible for the contraction of a newly identified highly cytotoxic T cell subset that strongly upregulates PD-1 in the late phase of hantavirus infection (81). Hantavirus-induced expression of PD-L1 and PD-L2 may contribute to the recently described protection of hantavirus-infected endothelial cells from cytotoxic attack by CD8+ T cells and NK cells (82). In line with this notion, antibody blockade of PD-L1 and PD-L2 on IFN-γ treated endothelial cells enhanced cytolytic activity of antigen-specific CD8+ T lymphocytes (8). Similarly, failure of the inhibitory PD-1/PD-L1 axis during hantavirus infection of vascular tissue may lead to unbalanced immunostimulation and immunopathology as proposed for inflammatory blood vessel diseases (83).

Despite checkpoint inhibition we observed bystander activation in a subset of T cells. Bystander activation of T lymphocytes represents a first line of antiviral defense and may contribute to hantavirus-induced immunopathogenesis. In line with his view, bystander T cells responding to dengue virus, another VHF virus, secrete IFN-γ (84). It has been reported that virus-induced bystander T cell activation bypasses control checkpoints such as Tregs (85). In accordance, we observed that hantavirus-induced bystander T cell activation is not prevented by PD-Ll/PD-L2 upregulation on monocyte-derived inflammatory DCs. This can be explained by the fact that bystander CD8+ T cell activation does not result in TCR-induced PD-1 upregulation. In contrast, TCR signaling induced by cognate antigen upregulates PD-1 expression on CD8+ T cells within the first 24 h during infection (86). This may ensure that virus-specific T cells are excluded from innate responses and differentiate into effector T cells of the adaptive immunity.

Hantavirus-induced bystander activation was strictly dependent on CD14+ cells. This may be explained firstly by the fact that monocyte-derived cells are needed for hantavirus infection in PBMCs. Secondly, CD86 is expressed almost exclusively on monocyte-derived cell types and we could show that CD86 was required for hantavirus-induced bystander activation. Thus, CD86 on hantavirus-infected DCs may activate heterologous CD8+ T cells through CD28. The importance of CD28 for bystander activation of CD8+ T cells has been previously described (87). It is unlikely that hantaviruses directly activate T lymphocytes through PRRs. However, previous reports demonstrated that inflammatory cytokines such as type I contribute to innate T cell activation (88–90). In accordance, we observed that blocking of type I IFN reduced bystander activation of CD8+ T cells upon hantavirus infection.

Many acute viral infections are known to trigger bystander activation of heterologous CD8+ T cells (91–93). Often CD8+ T cells specific for human herpesviruses contribute to the heterologous antiviral immune response (92). In line with this view, we observed activation of HCMV-specific memory CD8+ T cells in PBMCs from HCMV-seropositive patients after hantavirus infection. In fact, bystander activation of CD8+ T cells represent an early line of antiviral defense (94). Bystander activated CD8+ T lymphocytes control early pathogen load in virus-infected tissue by a NKG2D-dependent mechanism (95). In accordance with this concept, cytotoxic CD8+ T cells strongly expressing NKG2D were detected in the lung of hantavirus-infected patients (96). NKG2D ligands are upregulated by PRRs that sense viral replication (97). These include RIG-I, which has been shown to detect hantaviruses (30). Interestingly, a strong plasmablast response with reactivity against virus-unrelated antigens has recently been detected in patients with acute hantavirus pulmonary syndrome (98). Whether this heterologous B cell response has a pathogenic or protective role is unclear.

In conclusion, hantavirus-infected patients suffer from immunopathology in the face of immunosuppressive PD-L1 upregulated by hantaviral N protein and most likely hantavirus-induced TLR3 signaling. This apparent discrepancy could be explained by rapid cleavage and removal of PD-L1 from the surface of hantavirus-infected cells *in vivo*. In accordance, we detected large quantities of sPD-L1 in the serum of patients with hantavirus-associated disease. Moreover, the lack of opportunistic infections in these patients implies that PD-L1 does not globally suppress the immune system. Finally, early activation of heterologous CD8+ T cells during acute virus infections bypasses or overwhelms the inhibitory PD-1/PD-L1 axis and represents a means of eluding viral immune subversion at least in the short term (99).

## Author Contributions

MR designed research, performed all experiments, analyzed data, contributed to writing, and prepared figures. MA analyzed data and contributed to manuscript revision. JH provided serum samples from hantavirus-infected patients, contributed to manuscript revision and provided intellectual input. GS was involved in experiment conception, wrote the paper, analyzed data, provided intellectual input, and contributed to figure preparation.

### Conflict of Interest Statement

The authors declare that the research was conducted in the absence of any commercial or financial relationships that could be construed as a potential conflict of interest.
